# A systematic review of ongoing clinical trials in optic pathway gliomas

**DOI:** 10.1007/s00381-020-04724-1

**Published:** 2020-06-18

**Authors:** Ciaran Scott Hill, Sara Castro Devesa, William Ince, Anouk Borg, Kristian Aquilina

**Affiliations:** 1 Department of Neurosurgery, Great Ormond Street Hospital, London, UK; 2 UCL Cancer Institute, University College London, London, UK; 3 Department of Neurosurgery, Oxford University Hospital, Oxford, UK; 4 Ipswich Hospital, East Suffolk and North Essex NHS Trust, Health Road, Ipswich, IP45PD UK

**Keywords:** Optic, Visual, Pathway, Glioma, Trial, Systematic review

## Abstract

**Introduction:**

Optic pathway gliomas (OPGs), also known as Visual Pathway Gliomas, are insidious, debilitating tumours. They are most commonly WHO grade 1 pilocytic astrocytomas and frequently occur in patients with neurofibromatosis type 1. The location of OPGs within the optic pathway typically precludes complete resection or optimal radiation dosing, hence outcomes remain poor compared to many other low-grade gliomas. The aim of this systematic review was to formulate a comprehensive list of all current ongoing clinical trials that are specifically looking at clinical care of OPGs in order to identify trends in current research and provide an overview to guide future research efforts.

**Methods:**

This systematic review was conducted in line with the Preferred Reporting Items for Systematic Reviews and Meta-Analysis (PRISMA) guidelines. The Cochrane Controlled Register of Trials (CENTRAL) and ClinicalTrials.gov were searched. Inclusion and exclusion criteria were applied and final results were reviewed.

**Results:**

501 clinical trials were identified with the search strategy. All were screened and eligible studies extracted and reviewed. This yielded 36 ongoing clinical trials, 27 of which were pharmacological agents in phase I-III. The remaining trials were a mixture of biological agents, radiation optimisation, diagnostic imaging, surgical intervention, and a social function analysis.

**Conclusion:**

OPG is a complex multifaceted disease, and advances in care require ongoing research efforts across a spectrum of different research fields. This review provides an update on the current state of research in OPG and summarises ongoing trials.

## Introduction

Optic pathway gliomas (OPGs), also known as visual pathway gliomas, are insidious, debilitating tumours that account for 3–5% of all paediatric brain tumours. They are a subtype of low-grade glioma (LGG), most often World Health Organization (WHO) grade 1 juvenile pilocytic astrocytomas (JPAs), with a smaller proportion being pilomyxoid astrocytomas (PXAs) [1]. They are common in patients with neurofibromatosis type 1 (NF1), with up to 20% developing an OPG at a mean age of 4.5–5 years old [2]. OPGs most commonly arise at the chiasmatic-hypothalamic region but can arise anywhere along the optic pathway [3]. Their intimate relationship to the optic apparatus, hypothalamus, ventricular system and brain parenchyma can result in a variety of clinical sequelae including visual loss, endocrinopathies and hypothalamic dysfunction, developmental/neuropsychological disorders, hydrocephalus and focal neurological deficits. This complexity of symptomatology, and the close relationship to key structures make the treatment of OPG challenging. Unlike other JPAs, the location typically precludes complete surgical resection or optimum radiation dosing without incurring an often-unacceptable neurological cost. Furthermore, key aspects of their behaviour including their natural history and optimal management are incompletely understood. Tumour stabilisation, progression or regression can all occur unpredictably. OPG management is highly individualised. Many undergo a period of observation with serial imaging unless there is progressive growth or visual symptoms. Chemotherapy with a carboplatin/vincristine ‘Packer’ regimen is often the first line, although alternatives such as the TPCV (thioguanine, procarbazine, lomustine, vincristine) are sometimes used, except in NF1 patients due to the risk of secondary leukaemia [4]. Radiotherapy is typically avoided in younger children due to risks of toxicity wherever possible [5], although it remains an option in the setting of refractory, progressive disease or where visual loss is occurring despite systemic therapy [6, 7]. The role of surgical biopsy and debulking surgery remains controversial [8, 9]. OPGs require multidisciplinary care by neurosurgeons, neuro-oncologists, radiation oncologists, endocrinologists, ophthalmologists, pathology, neuropsychology, paediatricians, geneticists and a host of allied health professionals. This multifaceted care underlies the complexity of the disease. Advances in the understanding and treatment of OPG could come from a variety of sources, including novel or repurposed pharmacological agents, emerging biological agents and tumour vaccines, refinement of surgical approaches, diagnostic and therapeutic radiological developments or quality of life–focussed research [10–16]. The aim of this systematic review was to formulate a comprehensive list of all current ongoing clinical trials that were specifically looking at a clinical care of OPGs in order to identify trends in current research and provide an overview of the field to guide future research efforts. This review includes trials that are registered, and in any stage of recruitment or analysis, but not yet published. A summary of each of the ongoing clinical trials will be presented to facilitate a rapid review of the field.

## Methods

This systematic review was conducted in line with the Preferred Reporting Items for Systematic Reviews and Meta-Analysis (PRISMA) guidelines on April 10th, 2020 [17, 18]. Two clinical trial databases, the Cochrane Controlled Register of Trials (CENTRAL) and ClinicalTrials.gov, were searched. The search terms used to identify clinical trials were as follows: ‘(optic OR pathway OR visual OR opg OR chiasm OR midline OR hypothalamic OR hypothalamus OR neurofibromatosis OR NF1 OR pediatric OR paediatric) AND (glioma OR pilocytic OR astrocytoma OR pilomyxoid)’. Trials older than 2010 were excluded. Trial titles and abstracts were reviewed by two of the authors. Titles with no English language translation available, and duplicates, were removed. Exclusion criteria were applied; these included titles that were not related to OPG or to trials that would not include OPG as a subgroup based on their inclusion/exclusion criteria. Already-published studies were excluded; these articles were reviewed to ensure that the publications were final and complete, and not an interim analysis or partial publication, with further publication of trial results expected. Studies that had been withdrawn, abandoned or not updated in 3 years or more were also excluded. For identified trials where there had been no recent clinical trial update (in print or online) for > 3 years, contact was made with the principal investigator of the trial to establish progress and assess if the trial was still running. Two authors (CSH and AB) performed the search strategy independently and reviewed the final papers separately. Any conflicting findings were reviewed (by CSH, AB and WI) and a consensus agreed.

## Results

**Fig. 1:**
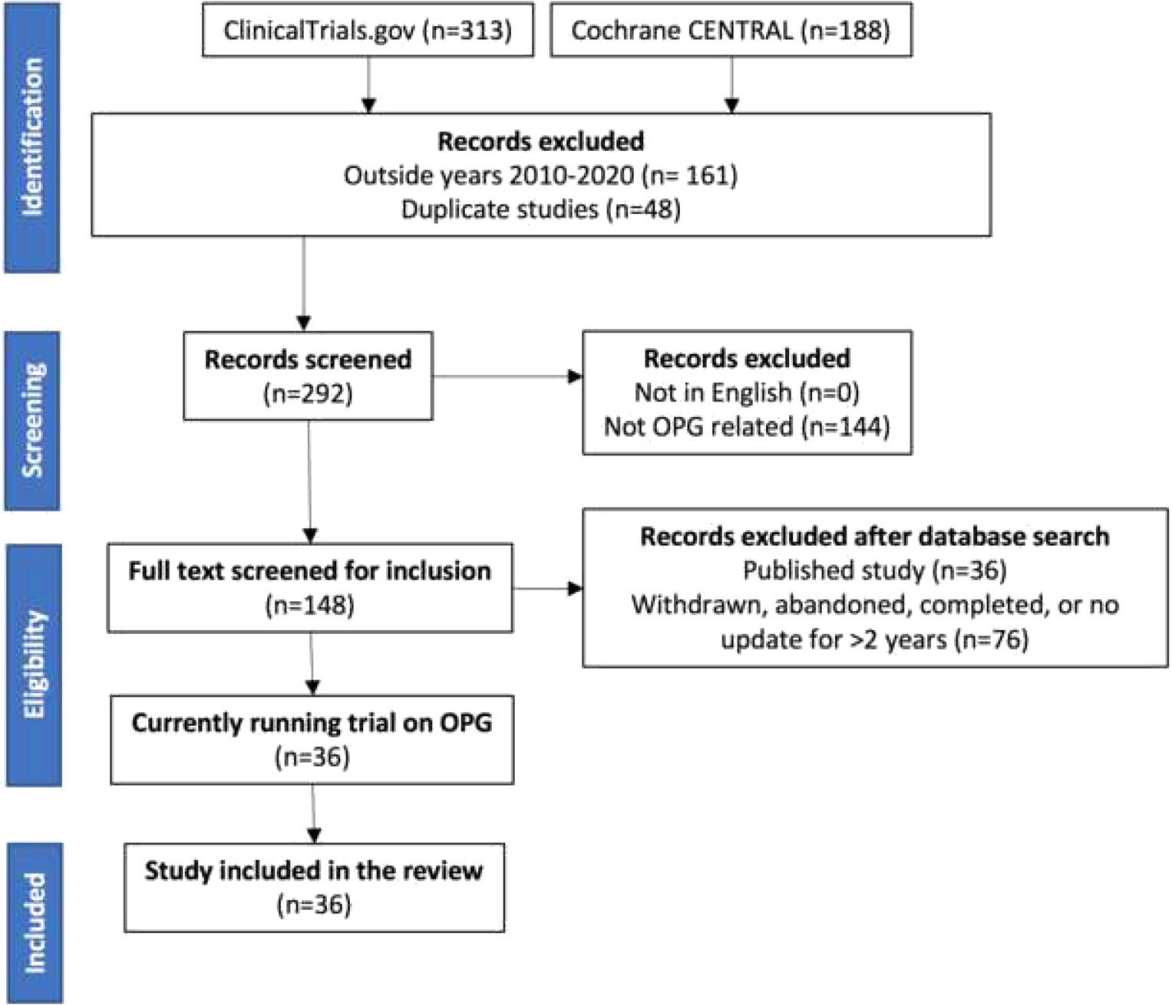
PRISMA process summary. This figure demonstrates article inclusion and exclusion across the various stages of review

Five hundred one clinical trials were identified with the search strategy described. After initial exclusions, 292 were screened. Thirty-six eligible studies were included in the final analysis. The identification, screening, eligibility and inclusion were conducted according to PRISMA guidelines. The strategy is summarised in Fig. 1.

After application of the PRISMA search strategy, we included 36 clinical trials in our final analysis.

A detailed summary of the stratified clinical trials included in the review is presented in Table 1.

**Table 1:** Clinical trials involving optic pathway glioma that are currently in progress

Trial name Registration number	Primary objective	Trial design	Estimated study completion	Sample size	Patient age	Intervention	Comparison	Primary outcome	Country (primary institution)
Phase 0/I pharmacological trials									
Pediatric Neuro-Oncology Consortium (PNOC)-002: Safety, Phase 0, and Pilot Efficacy Study of Vemurafenib, an Oral Inhibitor of BRAFV600E, in Children and Young Adults with Recurrent/Refractory BRAFV600E- or BRAF Ins T Mutant Brain Tumors NCT01748149	Assess safety and establish maximum tolerated and recommended phase II dose of vemurafenib in recurrent or refractory gliomas containing the BRAFV600E or BRAF Ins T mutation	Open label Non-randomised Single group Phase 0/I	December 2020	40	0–25 years	Drug: vemurafenib	Dose escalation	Maximum tolerated dose, toxicity, pharmacokinetics and objective response. Then intratumoural drug concentration	USA (UCSF)
Phase I pharmacological trials									
A Phase I Study of TAK-580 (MLN2480) for Children with Low-Grade Gliomas and other RAS/RAF/MEK/ERK Pathway Activated Tumors NCT03429803	Determine safety of treatment and appropriate dosage of TAK-580 in children and adolescent participants with LGG	Open label Non-randomised Single group Phase I	December 2024	53	1–25 years	Drug: TAK-580 (MLN2480)	N/A: safety analysis	Dose-limiting toxicity and progression-free survival	USA (Dana-Farber)
A Phase I Trial of Pomalidomide for Children with Recurrent, Progressive, or Refractory CNS Tumors NCT02415153	Determine best dosage of pomalidomide in treating younger patients with recurrent, progressive or refractory CNS tumours	Open label Non-randomised Single group Phase I	March 2020	42	3–20 years	Drug: pomalidomide	N/A: safety analysis	Maximum tolerated dose and recommended phase II dose, toxicity, pharmacokinetics	USA (NCI)
Sirolimus in Combination with Metronomic Therapy in Children with Recurrent and Refractory Solid Tumors: A Phase I Study NCT01331135	Investigate anti-tumour activity, optimal dosing and toxicity of sirolimus in combination with other chemotherapy in recurrent or refractory solid tumours	Open label Non-randomised Single group Phase I	April 2021	24	0–30 years	Drug: sirolimus	Dose escalation	Maximum tolerated dose and recommended phase II dose	USA (Emory)
Anti-Angiogenic Therapy After Autologous Stem Cell Rescue (ASCR) for Pediatric Solid Tumors NCT01661400	Assess feasibility and safety of using anti-angiogenic drugs after autologous stem cell transplant	Open label Non-randomised Sequential assignment Phase I	February 2020	12	6 months–21 years	Drugs: cyclophosphamide and thalidomide	Control versus cyclophosphamide versus thalidomide	Toxicity and best overall response	USA (WUSM)
Phase I/Ib Trial of Combined 5’Azacytidine and Carboplatin for Recurrent/Refractory Pediatric Brain and Solid Tumors NCT03206021	Determine maximum tolerated dose and initial efficacy of 5′azacytidine in combination with carboplatin	Open label Non-randomised Parallel assignment Phase I	July 2021	54	1–18 years	Drug: 5′azacytidine	Dose escalation then expansion	Maximum tolerated dose and feasibility of treatment	Canada (SickKids)
A Phase Ib Dose Escalation Study of Abemaciclib in Combination with Temozolomide and Irinotecan (Part A) and Abemaciclib in Combination with Temozolomide (Part B) in Pediatric and Young Adult Patients with Relapsed/Refractory Solid Tumors NCT04238819	Determine safety and effectivity of abemaciclib in combination with irinotecan and/or temozolomide	Open label Non-randomised Single group Phase I	February 2022	60	0–18 years	Drug: abemaciclib	Dose escalation then dose expansion	Toxicity, pharmacokinetics and response rate	USA (Eli Lilly)
A Phase 1 Study of Entinostat, an Oral Histone Deacetylase Inhibitor, in Pediatric Patients with Recurrent or Refractory Solid Tumors, Including CNS Tumors and Lymphoma NCT02780804	Determine the maximum tolerated and recommended dose of entinostat in children with recurrent or refractory solid tumours	Open label Non-randomised Single group Phase I	April 2022	36	1–21 years	Drug: entinostat	Dose escalation then dose expansion	Maximum tolerated dose, toxicity and pharmacokinetics	USA (NCI)
A Phase I Study of Mebendazole for the Treatment of Pediatric Gliomas NCT01837862	Determine maximally tolerated dose of mebendazole in combination with vincristine, carboplatin and temozolomide (for LGG) OR bevacizumab and irinotecan (for HGG)	Open label Non-randomised Parallel assignment Phase I	April 2020	36	1–21 years	Drug: mebendazole	Dose escalation then dose expansion	Toxicity and response rate of participants	USA (Janssen)
Phase I/II pharmacological trials									
A Phase 1 and Phase II and Re-Treatment Study of AZD6244 for Recurrent or Refractory Pediatric Low Grade Glioma NCT01089101	Phase I: maximum tolerated dose and recommended phase II dose of selumetinib. Phase II: objective response and disease stabilisation rates with selumetinib treatment	Open label Non-randomised Single group Phase I/II	December 2020	180	3–21 years	Drug: selumetinib (AZD6244)	Dose escalation then dose expansion	Toxicity and response rate of participants	USA (NCI)
Phase I/II Trial of Dabrafenib, Trametinib, and Hydroxychloroquine (HCQ) for BRAF V600E-mutant or Trametinib and HCQ for BRAF Fusion/Duplication Positive or NF1-associated Recurrent or Progressive Gliomas in Children and Young Adults NCT04201457	Phase I: maximum tolerated and recommended phase II dose of HCQ with dabrafenib and/or trametinib in young patients with recurrent or progressive glioma Phase II: response rate	Open label Non-randomised Parallel assignment Phase I/II	February 2025	75	1–30 years	Drug: hydroxychloroquine	Dose escalation then dose expansion	Maximum tolerated dose and response rate	USA (PBTC)
A Phase 2 Study of Trametinib for Patients with Pediatric Glioma or Plexiform Neurofibroma With Refractory Tumor and Activation of the MAPK/ERK Pathway. NCT03363217	Asses response rate of paediatric glioma and plexiform neurofibroma (PN) to trametinib	Open label Non-randomised Parallel assignment Phase I/II	June 2026	150	1 month–25 years	Drug: trametinib	N/A	Objective response rate	Canada (St Justine’s Hospital)
An Open-Label, Dose-Escalation, Phase I/II Study to Investigate the Safety, Pharmacokinetics, Pharmacodynamics and Clinical Activity of the MEK Inhibitor Trametinib in Children and Adolescents Subjects with Cancer or Plexiform Neurofibromas and Trametinib in Combination with Dabrafenib in Children and Adolescents with Cancers Harboring V600 Mutations NCT02124772	Establish safe dose and effectivity of trametinib monotherapy and in combination with dabrafenib in young patients	Open label Non-randomised Sequential assignment Phase I/II	December 2020	142	1 month–17 years	Drug: trametinib	Dose escalation	Safe dose, toxicity and response rate	USA (Novartis)
A Phase 1/2 Study of Lenvatinib in Combination with Everolimus in Recurrent and Refractory Pediatric Solid Tumors, Including CNS Tumors NCT03245151	Determine safety, optimal dose and response rate of lenvatinib combined with everolimus in paediatric patients with recurrent/refractory solid tumours	Open label Non-randomised Parallel assignment Phase I/II	May 2022	120	2–21 years	Drug: lenvatinib	Dose escalation then dose expansion	Maximum tolerated dose, recommended phase II dose, toxicity and response rate	USA (Eisai Inc)
A Paediatric Phase I/II Study of Intermittent Dosing of The Mek-1 Inhibitor Selumetinib In Children with Neurofibromatosis Type-1 And Inoperable Plexiform Neurofibroma and/or Progressive Optic Pathway Glioma Intermittent Dosing of Selumetinib in Childhood NF1 Associated Tumours (INSPECT) NCT03326388	Phase I: evaluate maximum tolerated dose of selumetinib Phase II: response rate to selumetinib in NF1 inoperable plexiform neurofibroma and OPG	Open label Non-randomised Single group Phase I/II	December 2023	30	3–18 years	Drug: selumetinib	Dose escalation	Response rate of participants measured with 3D volumetric analysis/2D assessment of tumour size	UK (GOSH)
Phase I Study of MEK162 for Children with Progressive or Recurrent Cancer and a Phase II Study for Children with Low-Grade Gliomas and other Ras/Raf/MAP Pathway Activated Tumors NCT02285439	Phase I: determine best dose and safety of MEK162 in children and adolescents Phase II: define response rate	Open label Non-randomised Parallel assignment Phase I/II	January 2021	120	1–18 years	Drug: MEK162	Dose escalation	Maximum tolerated dose and response rate	USA (CHLA)
Phase II pharmacological trials									
A Phase II, Open-Labeled, Multi-Center, Randomized Controlled Trial of Vinblastine +/- Bevacizumab for the Treatment of Chemotherapy-Naïve Children with Unresectable or Progressive Low-Grade Glioma (LGG) NCT02840409	Determine efficacy of adding bevacizumab to vinblastine in chemotherapy-naïve paediatric patients with progressive/unresectable LGG	Open label Randomised Parallel assignment Phase II	August 2026	150	6 months–18 years	Drug: bevacizumab	Vinblastine versus vinblastine plus bevacizumab	Response rate	Canada (SickKids)
Phase II Open-label Global Study to Evaluate the Effect of Dabrafenib in Combination with Trametinib in Children and Adolescent Patients with BRAF V600 Mutation Positive Low Grade Glioma (LGG) or Relapsed or Refractory High Grade Glioma (HGG) NCT02684058	Investigate effect of dabrafenib combined with trametinib in young patients with LGG BRAF V600 positive or relapsed or refractory HGG	Open label Non-randomised Single group Phase II	November 2021	142	1–17 years	Drug: dabrafenib	Dabrafenib and trametinib versus carboplatin and vincristine	Overall response rate	USA (Novartis)
NCI-COG Pediatric MATCH (Molecular Analysis for Therapy Choice) - Phase 2 Subprotocol of BVD-523FB (Ulixertinib) in Patients with Tumors Harboring Activating MAPK Pathway Mutations NCT03698994	Assess efficacy of ulixertinib in patients with mutations in the MAPK signalling pathway	Open label Non-randomised Single group Phase II	December 2025	49	1–21 years	Drug: ulixertinib	N/A	Objective response rate	USA (NCI)
Memantine for Prevention of Cognitive Late Effects in Pediatric Patients Receiving Cranial Radiation Therapy for Localized Brain Tumors: A Pilot Study NCT03194906	Estimate participation rate in a study of memantine as a neuroprotective agent, rate of medication adherence and completion of cognitive assessments	Double blind Randomised Placebo controlled Parallel assignment Phase II	January 2021	50	6–21 years	Drug: memantine	Memantine versus placebo	Percentage who participate, complete 12 weeks of therapy and a minimum of 3 cognitive assessments	USA (St. Jude)
Phase II Study of Everolimus for Recurrent or Progressive Low-grade Gliomas in Children NCT01734512	Determine efficacy of everolimus in children with recurrent or progressive LGG	Open label Non-randomised Single group Phase II	January 2028	66	3–21 years	Drug: everolimus	N/A	Progression-free survival	USA (UCSF)
A Phase II Trial of Poly-ICLC in the Management of Recurrent Pediatric Low Grade Gliomas NCT01188096	Determine safety and effectivity of poly-ICLC in young patients with LGG	Open label Non-randomised Single group Phase II	July 2019	23	0–21 years	Drug: poly-ICLC	N/A	Overall response rate	USA (UCSD)
Pilot Study of the MEK1/2 Inhibitor Selumetinib (AZD6244 Hydrogen Sulfate) for Adults with Neurofibromatosis Type 1 (NF1) and Cutaneous Neurofibromas (CNF) NCT02839720	Establish if selumetinib can result in volume decrease of cutaneous neurofibromas	Open label Non-randomised Single group Phase II	December 2021	24	18+ years	Drug: selumetinib	N/A	Change in tumour size	USA (NCI)
A Phase II Study of Pegylated Interferon alfa-2b in Children with Recurrent or Refractory and Radiographically or Clinically Progressive Juvenile Pilocytic Astrocytomas & Optic Pathway Gliomas NCT02343224	Response rate to pegylated interferon	Open label Non-randomised Single group Phase II	December 2021	20	3–25 years	Drug: pegylated interferon alfa-2b	N/A	Response rate of participants from baseline to 2 years	USA (Emory)
A Phase II Randomized Trial of Lenalidomide (NSC # 703813) in Pediatric Patients with Recurrent, Refractory or Progressive Juvenile Pilocytic Astrocytomas and Optic Pathway Gliomas NCT01553149	Response rate (complete or partial) to low- or high-dose lenalidomide	Open label Randomised Parallel assignment Phase II	June 2020	80	0–21 years	Drug: lenalidomide	Low-dose or high-dose lenalidomide	Objective best response rate	USA (NCI)
Phase III pharmacological trials									
A Phase 3 Randomized Study of Selumetinib Versus Carboplatin/Vincristine in Newly Diagnosed or Previously Untreated Neurofibromatosis Type 1 (NF1) Associated Low-Grade Glioma NCT03871257	Assess selumetinib compared with treatment with carboplatin/vincristine (CV) for participants with NF1-associated low-grade glioma, and to assess if selumetinib is better than CV in improving vision in participants with LGG of the optic pathway	Open label Randomised Parallel assignment Phase III	May 2027	290	2–21 years	Drug: selumetinib	Selumetinib versus carboplatin and vincristine	Event-free survival and number of participants with visual improvement	USA (NCI)
A Phase 3 Randomized Non-Inferiority Study of Carboplatin and Vincristine Versus Selumetinib (NSC# 748727) in Newly Diagnosed or Previously Untreated Low-Grade Glioma (LGG) Not Associated with BRAFV600E Mutations or Systemic Neurofibromatosis NCT04166409	Determine efficacy of treatment with selumetinib compared with carboplatin and vincristine in untreated LGG (not NF1 or BRAFV600E associated)	Open label Randomised Parallel assignment Phase III	December 2026	200	2–12 years	Drug: selumetinib	Selumetinib versus carboplatin and vincristine	Event-free survival	USA (NCI)
Biological therapy trials									
A Phase I Study of Ad-RTS-hIL-12, an Inducible Adenoviral Vector Engineered to Express hIL-12 in the Presence of the Activator Ligand Veledimex in Pediatric Brain Tumor Subjects NCT03330197	Assess safety and tolerability of intratumoural Ad-RTS-hIL-12 injection in combination with oral veledimex	Open label Non-randomised Sequential single groups Phase I	March 2021	24	0–21 years	Biological: Ad-RTS-hIL-12	N/A: safety analysis	Dose-limiting toxicity and safety of treatment	USA (Ziopharm)
Phase 1 Study of B7-H3-Specific CAR T Cell Locoregional Immunotherapy for Diffuse Intrinsic Pontine Glioma/Diffuse Midline Glioma and Recurrent or Refractory Pediatric Central Nervous System Tumors NCT04185038	Assess safety and feasibility of B7H3-specific CAR T cell administration via indwelling catheter into the tumour resection cavity or ventricular system in DIPG, DMG and recurrent or refractory CNS tumours	Open label Non-randomised Parallel assignment Phase I	May 2041	70	1–26 years	Biological: B7H3-specific CAR T cell	N/A: safety analysis	Successful delivery, toxicity and response rate	USA (Seattle Children’s)
Radiotherapy-based trials									
A Phase II Study of Proton Radiotherapy for Pediatric Brain Tumors Requiring Partial Brain Irradiation: An Assessment of Long Term Neurocognitive, Neuroendocrine an Ototoxicity Outcomes NCT01288235	Determine if proton radiotherapy has reduced long-term neuroendocrine and neurocognitive impact compared with standard radiation	Open label Non-randomised Single group Phase II	September 2022	100	1–25 years	Radiation: proton radiotherapy	Standard radiation versus proton radiotherapy	Endocrine dysfunction and neurocognitive sequelae	USA (MGH)
A Phase II Study of Hippocampal-Avoidance Using Proton Therapy in Low-Grade Glioma NCT04065776	Determine feasibility of reducing radiation therapy doses to the hippocampi using proton therapy	Open label Non-randomised Single group Phase II	May 2023	74	6–21 years	Radiation: hippocampal-avoidance proton therapy	N/A	Percentage of plans meeting first or second dose constraints	USA (St. Jude)
Diagnostic imaging trials									
Developing Fast Pediatric Imaging NCT03761121	Development of improved MRI technologies to better measure brain tumour tissue volume	Open label Non-randomised Parallel assignment	September 2021	300	All	Other: Wave-CAIPI (controlled aliasing in parallel imaging)	N/A	Validation of Wave-CAIPI as a diagnostic tool	USA (Dana-Farber)
Pilot Study of [18F] Fluorodeoxyglucose Positron Emission Tomography- Magnetic Resonance Imaging (FDG-PET-MRI) in CNS and Extra-CNS Tumors of Patients with Neurofibromatosis-1 (NF1) NCT01800032	Acquire preliminary data and report differences in FDG-avidity in patients with NF1-associated optic glioma and plexiform neurofibroma	Observational Prospective cohort study (pilot)	December 2019	30	6+ years	Device: FDG-PET-MRI	Progressive versus non-progressive disease	Comparison of FDG-avidity between progressive and non-progressive lesion/disease	USA (Lineberger Centre)
Miscellaneous trials (genetic, social function, image-guided thermotherapy)									
Frameshift Peptides of Children with Neurofibromatosis Type 1 (NF1) and Either Low-Grade Gliomas or Plexiform Neurofibromas NCT04212351	Establish if children and young adults with NF1 and LGGs or plexiform neurofibroma have a specific frameshift peptide profile for potential development of a disease-specific vaccine	Observational Prospective cohort study	April 2021	60	0–30 years	Genetic: frameshift array blood sample test	Active LGG versus plexiform neurofibroma, versus neither	Frameshift peptide protein profile in patients with NF1 and those who develop LGGs versus plexiform neurofibromas	USA (CNRI)
Components of Social Functioning in Survivors of Pediatric Brain Tumors NCT03361033	Observe social and neurocognitive functioning in paediatric survivors of brain tumours	Observational Prospective cohort study	February 2021	80	8–12 years	N/A	N/A	Predictors of social, cognitive and neurocognitive abilities on social interaction	USA (St. Jude)
A Pilot Study of Using MRI-Guided Laser Heat Ablation to Induce Disruption of the Peritumoral Blood Brain Barrier to Enhance Delivery and Efficacy of Treatment of Pediatric Brain Tumors NCT02372409	Assess and establish the window of maximal blood–brain barrier disruption post laser ablation	Open label Non-randomised Parallel assignment Phase I	October 2020	12	3–21 years	Device: MRI-guided laser ablation	MRI-guided laser ablation versus MRI-guided laser ablation plus doxorubicin and etoposide	Progression-free survival, overall survival and quality of life	USA (Washington University)

Out of the 36 clinical trials assessed, 27 were pharmacological studies. There was one phase 0 trial, 8 phase 0/I drug trials, 7 mixed phase I and phase II, 9 phase II, and 2 phase III trials. There were 2 trials focussed on biological agents, 2 based on optimising radiation therapy, 2 on diagnostic neuroimaging, one on imaging-guided thermotherapy, one on genetic testing and one on social functioning.

## Discussion

Systematically reviewing the ongoing clinical trials is important to update clinicians on potential new therapies, avoid duplication of research and identify research trends and developing areas of study to stimulate new investigation. As might be expected, the main focus of ongoing research studies in OPG is related to pharmacological agents. Twenty-seven out of the 36 identified research trials were drug trials. Such investigations are necessary to establish safety and efficacy of all new drugs and are essential to determine if these agents should be adopted or discarded. Many of the new agents were related to the BRAF/MEK/ERK or RAS pathways. A summary of the pharmacological agents under investigation is presented in Table 2.

**Table 2:** Pharmacological agents under investigation as potential therapies in optic pathway gliomas

Drug/intervention	Proposed mechanism of action
BRAF/MEK/ERK pathway targeting agents The MAPK/ERK signalling pathway is a key regulator of biological growth, proliferation and differentiation. The MAPK/ERK pathway is often upregulated in a variety of tumour types including glioma. The BRAFV600E mutation leads to constitutive overactivation of BRAF which is a driver of tumourigenesis in BRAFV600E mutant low-grade gliomas [27–29].	
Vemurafenib/dabrafenib	Selective type 1 B-Raf competitive small molecule enzyme inhibitors. Selectively recognises and binds the ATP-binding domain of BRAFV600E mutants. This interrupts the B-Raf/MEK step on the B-Raf/MEK/ERK pathway that is a driver of tumourigenesis in BRAFV600E mutant LGGs. Vemurafenib has shown some promise in the treatment for BRAFV600E mutant LGGs [30].
Tak580	Oral pan-Raf kinase inhibitor. A single-patient study demonstrated apparently good tolerance in a patient with refractory LGG ahead of a larger planned clinical trial [31].
Ulixertinib	An ERK1/2 inhibitor. Blocking ERK-mediated signal transduction may inhibit ERK-dependent tumour cell proliferation and survival.
RAS pathway targeting agents The RAS/MAPK pathway is important in the control of cancer cell growth and proliferation. The NF1 product neurofibromin functions as a negative regulator of RAS activity. RAS overactivity in patients with NF1 drives tumourigenesis. The MEK pathway is hyperactivated in NF1-deficient astrocytes and this drives NF1-associated optic glioma growth. Hence, the rationale for sustained MEK pharmacologic blockade in attenuating NF1-deficient astrocytes and NF1 optic glioma volume and proliferation [32]. The development of agents that target RAS signalling and associated pathways implicated in the pathogenesis of neurofibromas has led to clinical trials of various new pharmacological agents.	
Selumetinib/trametinib	Selective MEK inhibitor. A phase I study assessing selumetinib dose tolerance and pharmacokinetics in NF1-related plexiform neurofibromas was conducted by Dombi et al. who demonstrated benefit in inoperable plexiform neurofibromas without excess toxicity [33]. A further phase II study in relapsed LLG that included OPG showed sustained partial therapeutic response [34].
MEK162 (binimetinib)	Orally available inhibitor of MAPK2.
Anti-angiogenic agents	
Bevacizumab	A humanised monoclonal antibody directed against VEGF, this inhibition leads to a reduction in microvascular growth of tumour blood vessels and thus limits the blood supply to tumour tissues. There are several reports of the benefit of anti-VEGF drugs in uncontrolled case series [35–38].
Lenvatinib	Multireceptor TK inhibitor with preferential anti-angiogenic capacity. It inhibits VEGF receptor family 1-3, FGFR family 1-4, PDGFRa, TK receptor and RET. As a result, it may modulate the tumour microenvironment and anti-tumour responses [39].
Thalidomide	An anti-angiogenic agent. The mechanism of anti-angiogenic action is not fully known [40]. Its use has been investigated in the care of high-grade glioma [41].
Lenalidomide	An analogue of the anti-angiogenic agent thalidomide. It has demonstrated a significant anti-tumour activity in patients with multiple myeloma and myelodysplastic syndrome. Phase I studies in adults and children demonstrated tolerability but was associated with thromboembolic events and myelosuppression [42–44]. Antagonistically, the use of lenalidomide has been associated with secondary primary high-grade astrocytoma development in multiple myeloma [45].
Pomalidomide	An analogue of the anti-angiogenic agent thalidomide. It failed to demonstrate benefit in a phase II clinical study as monotherapy for children and young adults with recurrent or progressive primary brain tumours [46].
Miscellaneous pharmacological agents	
Mebendazole	Methyl N-[6-(benzoyl)-1H-benzimidazol-2-yl] carbamate. A benzimidazole anti-helminthic used to treat a variety of worm infections and for echinococcosis (hydatid disease). Mebendazole binds to tubulin subunits preventing polymerisation. Tubulin is a key molecule in cell division and has been proposed as an anti-cancer target. Other potential targets of mebendazole include the Hh signalling pathway, and angiogenesis through VEGF inhibition [47]. Repurposing of mebendazole has been suggested in various tumours and it has been previously trialled in preclinical models of glioblastoma with some success [48–50].
Entinostat	Entinostat is a synthetic benzamide HDAC type 1 and III inhibitor. It is orally bioactive. Its proposed mode of action is to regulate chromatin structure and through histone deacetylation control epigenetic gene transcription. Other mechanisms of effect may include induction of reactive oxygen species leading to mitochondrial damage and inducing cell cycle arrest [51, 52].
Pegylated interferon alfa-2b	Pegylated interferon α-2b is a member of the interferon family; these are glycoproteins with anti-cytokine effects that have documented immune-modulating and anti-proliferative effects. One phase II study is examining the effect of pegylated interferon alfa-2b in a child with recurrent or refractory/progressive JPA and OPG in children. Studies have shown that interferon alfa-2b is tolerable and may delay progression in DIPG [53].
Abemaciclib	Reversible small molecular CDK inhibitor. Some gliomas have been shown to overexpresses cyclin D1, which in turn increases activity of CDK4 and CDK6; these enzymes phosphorylate (and therefore deactivate) retinoblastoma protein. Deactivating retinoblastoma protein leads to excess cellular growth by loss of cell cycle control at the G1 to S phases. Abemaciclib has demonstrated some effect in slowing growth in preclinical glioblastoma models [54].
Hydroxychloroquine	Various possibilities including modulation of autophagy, cellular metabolism or direct chemotoxic effects [55].
Sirolimus/everolimus	First- and second-generation mTOR inhibitors. The mTOR pathway is important in control of cellular growth. These drugs have been trialled in giant cell astrocytomas as part of tuberous sclerosis complex, and everolimus is currently used in some cases that are not amenable to surgical resection [56, 57].
Memantine	An NMDAR1 antagonist thought to have anti-tumour and neuroprotective effects. It has been studied in glioblastoma.
Poly-ICLC	A synthetic double-stranded RNA complex. Poly-ICLC is a ligand for toll-like receptor-3 and MDA-5 that can activate immune cells (including dendritic cells and natural killer cells) and therefore act as a stimulating immunomodulating agent to induce tumour cell killing [58].
Cyclophosphamide	A chemotherapeutic agent. Cyclophosphamide forms an active metabolite phosphoramide mustard in cells with low levels of ALDH, as is the case in some tumour cells. The phosphoramide mustard forms irreversible DNA interstrand cross-links that trigger tumour cell apoptosis. It has been shown to enhance glioma virotherapy by inhibiting innate immune responses [59–61].
5′Azacytidine	An inhibitor of DNA methylation. This may be beneficial in LGGs with hypermethylation as part of their genetic phenotype [62].

*BRAF* rapidly accelerated fibrosarcoma gene B (B-Raf = protein), *CDK* cyclin-dependent kinase, *DIPG* diffuse intrinsic pontine glioma, *ERK* extracellular signal–regulated kinases, *FGFR* fibroblast growth factor receptor, *HDAC* histone deacetylase, *Hh* hedgehog, *LGG* low-grade glioma, *MAPK* selective mitogen-activated protein kinase, *MEK* selective mitogen-activated protein kinase, *mTOR* mammalian target of rapamycin, *NF1* neurofibromatosis 1, *NMDAR N*-methyl-d-aspartate receptor, *OPG* optic pathway glioma, *PDGFRa* platelet-derived growth factor receptor alpha, *RET* rearranged during transfection receptor, *Poly-ICLC* polyinosinic-polycytidylic acid-poly-l-lysine carboxymethylcellulose, *TK* tyrosine kinase, *VEGF* vascular endothelial growth factor

There were 5 ongoing trials into the same pharmacological agent selumetinib, and 2 looking at trametinib; this raises a question as to whether a collaborative approach would allow data sharing and ensure a common data element and consensus reporting. The included trials were dominated by North American centres and pharmaceutical companies. We did not identify any trials run from a low- or middle-income country (LMIC) despite reports that up to 80% of all paediatric cancer patients occur in these countries. Potential reasons for the lack of clinical trials in LMIC include a lack of specialised centres, equipment and staff, and a lower priority of cancer for healthcare planning strategies [19–21]. There were a small number of trials looking at non-pharmacological diagnostics, therapeutics and assessment social functioning relating to OPG. These are summarised in Table 3.

**Table 3:** Ongoing non-pharmacological trials related to optic pathway glioma

Object of investigation	Explanation
Non-pharmacological therapeutics	
Ad-RTS-hIL-12	An inducible adenoviral (Ad) vector encoding human pro-inflammatory cytokine interleukin-12 (hIL-12) p70 transgene. This is under the transcriptional control of the RheoSwitch Therapeutic System (RTS) and can be activated by administration of the proprietary diacylhydrazine-based activator ligand veledimex. Activation and transcription of IL-12 is proposed to have immunomodulating and anti-neoplastic activities [63].
B7-H3-specific CAR T cell	Chimeric antigen receptor (CAR) T cell therapy has emerged as a potential treatment in a range of cancers. CAR T cells are synthetic molecules composed of antibody binding domains connected to T cell activating domains and co-stimulatory domains. This allows highly specific tumour antigen recognition and subsequent T cell activation. The B7-H3 antigen is a transmembrane protein that is overexpressed in a range of tumours, and so manufacture of CAR T cells targeted to B7-H3 may be effective in a range of solid cancers including glioma [64, 65].
Proton radiotherapy	There were two studies identified that relate to proton radiotherapy. Protons are high-energy subatomic particles that when accelerated can be used to deliver focussed ionising radiation to a tumour in order to damage its DNA—killing tumour cells and inhibiting growth. One study is focussed on assessing long-term neurocognitive, neuroendocrine and ototoxicity outcomes. The other is assessing the capacity of proton beam therapy to successfully avoid hippocampal irradiation in LGG.
MRI-guided laser heat ablation	MRI-guided laser ablation (MLA) is a minimally invasive laser surgery technique that involves a scalp incision and a small burr hole through which a laser probe is inserted into the centre of a brain tumour under MRI guidance. The probe delivers hyperthermic ablation to the tumour which can destroy tumour tissue and also disrupts the tumour blood–brain barrier potentially enhancing the delivery of chemotherapeutic agents [66].
Frameshift peptides	A frameshift mutation is caused by insertion of deletion of a nucleotide sequence into DNA that creates a ‘frameshift’ as it is not a multiple of 3 (the number of nucleotides in a codon). This frameshift alters the whole DNA sequence and results in an altered gene and subsequent protein product. These ‘frameshift peptides’ can act as new epitopes that are potential targets for tumour vaccines. The study NCT04212351 proposes to determine if NF1-associated LGGs have a specific frameshift peptide protein profile that could be targeted with a specific vaccine [67].
Imaging diagnostics	
Fast paediatric imaging with Wave-CAIPI	Wave-CAIPI (controlled aliasing in parallel imaging) is an MRI-based technology that is faster than conventional MRI imaging. Parallel imaging works by acquiring a reduced amount of *k*-space data with an array of receiver coils. Research is underway to see if it can provide equivalent information to the current gold-standard MRI [68, 69].
Positron emission tomography	18F-Fluorodeoxyglucose (FDG) positron emission tomography (PET) is a nuclear imaging modality that identifies increased glucose uptake in tissues. FDG-PET can be fused with MRI to improve the identification of high-grade features in gliomas [70].
Psychological assessment	
Social functioning	Children with brain tumours are at risk for a number of late psychological effects, including neurocognitive and social deficits. These can be assessed with psychological questionnaires [16].

### Pending trials

The authors are aware of a further relevant clinical trial that is yet to start, the LOw Grade Glioma In Children (LOGGIC) study. LOGGIC will compare the effect of standard chemotherapy versus MEK inhibitor (trametinib) as first-line agents in paediatric low-grade glioma. The outcome measures will include a child’s quality of life, including visual and neurological function. The LOGGIC trial will be run by the European Society for Paediatric Oncology (SIOPE). As with any emerging field, this is likely to be just one of several trials not yet registered on public databases.

### Unanswered questions

This systematic review highlights the dominant pharmacologic-centric nature of current OPG clinical trial research. There are several important areas of OPG care that remain clouded in uncertainty and controversy and are not currently being addressed by the ongoing clinical trials we identified in this study. A key outstanding question is regarding the natural history of OPG and how we can predict outcomes. We have a paucity of understanding of the natural history of OPGs, and we lack tools to predict their clinical course and long-term outcomes. Furthermore, it is controversial which outcome measure should be used. Commonly used oncological outcome measures such as overall survival and (radiological) progression-free survival may not be the most appropriate for the study of OPGs; alternatives include visual function, endocrine/hypothalamic dysfunction and quality-of-life measures. While one trial identified in this review relates to social functioning, this remains a poorly examined area. Hypothalamic dysfunction is a key component of the OPG disease that is often neglected in clinical studies and can be challenging to quantify. The hypothalamic consequences of existing and emerging therapies need examination. Questions remain regarding the optimal therapeutic management. Although general consensus statements exist, there remains uncertainty around the optimal surgical strategy. The timing and nature of the surgical approaches to OPGs have not been subjected to rigorous trial data. Surgical options include biopsy, partial/subtotal resection and radical resection [13]. Furthermore, the optimal timing of chemotherapy/radiotherapy initiation and the influence this has on overall outcomes are not fully known. The focus of current research, including all trials identified in this study, is on recurrent/refractory OPGs rather than new diagnosis. The long-term efficacy of various treatments for newly diagnosed OPGs is unknown. Optic pathway gliomas are typically treated as a single disease. However, it may be important to stratify and subclassify based on factors such as age (infantile versus juvenile) and based on molecular/genetic subtyping. An example of this is stratification into syndromic/NF1-related and non-syndromic/non-NF1-related OPGs. These patients have different anatomical predilections, their natural histories can vary and the optimal therapies may be different [22–24].

### Limitations

We found few trials that were specifically focussed on OPGs as a distinct tumour subtype. As OPGs are a subset of LGG, they may be bundled into trials under umbrella terms like ‘LGG’ or ‘glioma’ or ‘JPA/PXA’. We not only developed a search strategy aimed at capturing trials that had specific reference to OPG (with the search terms; optic/visual/pathway/chiasm/midline/hypothalamus/hypothalamic) but also included terms to capture trials that may include OPGs without specific reference to them, e.g. by using terms (Neurofibromatosis/NF1/paediatric/paediatric) combined with generic blanket terms (glioma/astrocytoma/pilocytic/pilomyxoid). Where appropriate, we screened the trial protocols and inclusion/exclusion criteria to determine if OPGs might be included in the trials. As with any systematic review, there is always a risk that our search strategy missed a relevant trial. This risk is higher with a disease like OPG that is a subcategory of a wider disease. In order to try and minimise this danger, we used a reproducible search strategy that conformed to PRISMA guidelines. We elected to include all trials that could recruit OPGs; however, the vast majority are not focussed on OPGs but on LGG in general. We ensured that all included trials would allow OPGs as part of their criteria, but we cannot be sure that they will eventually recruit patients with this pathology in representative numbers, or if they will examine the data adequately to allow subgroup analysis of this pathology.

## Conclusion

OPG are a debilitating childhood cancer that causes a significant burden of suffering. Our understanding of this disease is limited and we lack effective treatments and clear management consensus [1, 25]. This manuscript details a systematic review of current ongoing trials in OPG. We find that from an initial yield of 501 hits, 36 ongoing trials fulfilled criteria for inclusion. The majority of these are studies or pharmacological agents, mostly phase I or II. OPG is a complex multifaceted disease, and advances in care are likely to require ongoing research efforts across a spectrum of different research fields [26]. Studies investigating surgical interventions and quality of life were notably absent. This review provides an update on the current state of research in OPG and highlights the main agents under investigation. We hope that this updates and stimulates clinicians and research scientists to engage with this important topic.

## Abbreviations

CENTRAL: Cochrane Controlled Register of Trials
FDA: Food and Drug Administration
GOSH: Great Ormond Street Hospital
HDAC: Histone deacetylase
HGG: High-grade glioma
JPAs: Juvenile pilocytic astrocytomas
LGG: Low-grade glioma
LMIC: Low- or middle-income country
MRI: Magnetic resonance imaging
NCI: National Cancer Institute
NF: Neurofibromatosis
OPG: Optic pathway glioma
PXAs: Pilomyxoid astrocytomas
PRISMA: Preferred Reporting Items for Systematic Reviews and Meta-Analysis
RCT: Randomised controlled trial
WHO: World Health Organization
WHO ICTRP: World Health Organization International Clinical Trials Registry Platform
